# Selection of superior genotypes of Indian jujube (*Ziziphus mauritiana* Lamk.) as revealed by fruit‐related traits

**DOI:** 10.1002/fsn3.2721

**Published:** 2022-01-17

**Authors:** Farhad Mirheidari, Ali Khadivi, Abdolvahid Saeidifar, Younes Moradi

**Affiliations:** ^1^ Department of Horticultural Sciences Faculty of Agriculture and Natural Resources Arak University Arak Iran; ^2^ Ministry of Agriculture Jihad Sistan‐va‐Baluchestan Iran

**Keywords:** gene pool, morphological variation, quality, skin color, superior genotype

## Abstract

The nutritional and medicinal benefits of *Ziziphus mauritiana* Lamk. have led to its attention. Here, morphological and pomological diversity of this species was investigated. Most of the characters recorded showed considerable differences among the genotypes studied. The range of ripening data was from mid‐February to mid‐March. Fruit weight ranged between 15.68 and 33.62 g with an average of 24.17. Strong diversity was observed among the genotypes in terms of fruit skin ground color, ranging from light green to orange. There were significant correlations between some characters especially between the traits related to fruit size. Principal component analysis (PCA) classified the traits into 12 main components, justifying 75.07% of the total variance. The studied genotypes were grouped into two main clusters, indicating strong diversity among them. The present information might be used to choose the genotypes with the desired traits. Twenty‐one genotypes were promising because of high values of fruit weight, fruit taste, fruit skin color, and fruit quality, and thus, they can be recommended for direct cultivation and also to be used in breeding programs. The genotypes with superior traits can be further used for improvement through selection and hybridization to get desired traits.

## Graphical abstract

Twenty‐one genotypes of *Ziziphus mauritiana* Lamk. were promising because of high values of fruit weight, fruit taste, fruit skin color, and fruit quality, and thus, they can be recommended for direct cultivation and also be used in breeding programs.

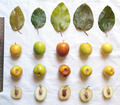

## 1 INTRODUCTION

Indian jujube (*Ziziphus mauritiana* Lamk., Rhamnaceae family) is rich in nutritional and medicinal properties. Tropical regions of South Asia, Australia, and Africa are its main distribution centers (Pasternak et al., 2009). In the arid and semi‐arid regions, this plant is multipurpose and also can be used to prevent soil erosion (Gupta, 2018; Pareek, 2001). It has been confirmed that Indian jujube has high tolerance to salinity, drought, flooding, and withering (Grice, 1997). The medicinal and nutritional benefits of this plant have led to its attention. Vitamins are detected in its fruits. It is used as a sedative and anticancer. Also, it is suitable as wound healer and also against asthma (Ashraf et al., 2015; Hudina et al., 2008; Mishra et al., 2011; Nyanga et al., 2013). The antioxidant activity of its fruits (Gupta, 2018; Okala et al., 2014), seeds (Bhatia & Mishara, 2009), and leaf (Dahiru & Obidoa, 2007; Gupta, 2018) have been detected.

Self‐incompatibility and cross‐pollination have increased genetic variation of Indian jujube. There are superior genotypes in *Z*. *mauritiana* with high values for commercial characters that are cultivated in the orchard via asexual propagation methods (Devanshi et al., 2007). The breeding programs of plants need suitable genetic variation. Evaluation of genetic variability is essential for efficient application in breeding programs as well as for the implementation of conservation strategies. Lack of awareness of the genetic diversity and distribution of a plant species is one of the main obstacles to germplasm management. The study of genetic diversity is essential to identify distinct and superior genotypes, to explain the relationship between genotypes, and to manage and use germplasm properly (Awasthi et al., 2009; Hurtado et al., 2012).

The selection does not make diversity in germplasm because it creates small populations for breeders so that they can find ideal genotypes (Pommer, 2012). Choosing the suitable cultivar for successful cultivation with acceptable yield in specific areas is very important (Aulakhet al., 2000). The interaction between genetics and environment has an important function in expressing qualitative and also quantitative traits of a genotype. Morphological descriptions are still the first step in assessing the phenotypic diversity of plants helping breeders to identify genotypes with desired traits (Jannatabadi et al., 2014; Khadivi‐Khub et al., 2014).

The *Z. mauritiana* is distributed in the southern parts of Iran, but little research has been done on this species. Therefore, phenotypic diversity of this important species was performed for the selection of superior genotypes for cultivation. The obtained information can be also useful in the protection and management of the genotypes.

## 2 MATERIALS AND METHODS

### 2.1 Plant material

Here, 119 genotypes of *Z. mauritiana* were chosen from different areas of Sistan‐va‐Baluchestan province in the south of Iran, and then, their morphological and pomological variation was evaluated. The geographical characteristics of the sites studied are shown in Table 1. The selected genotypes are the most important cultivated trees in the study areas. The orchard management operations, including nutrition, irrigation, and pest and disease control, were performed regularly and uniformly for the genotypes.

**TABLE 1:** Geographical description for collection sites of *Z. mauritiana* genotypes investigated from Sistan‐va‐Baluchestan province in Iran

No.	Area	Longitude (E)	Latitude (*N*)	Height (m)
1	Molakarim	61°25′48″	26°14′04″	252
2	Talsar	61°59′08″	26°12′14″	274
3	Mandust	61°57′44″	26°14′25″	254
4	Soldan	61°34′06″	26°14′46″	235
5	Doshanbechah	61°45′18″	26°18′14″	250

### 2.2 The recorded characteristics

Phenotypic variability of the genotypes was investigated based on 44 quantitative and qualitative characters related to tree, leaf, and fruit (Table 2). The 50 mature leaves and 50 mature fruits were used to record the related characters. A digital caliper was used to measure the characters related to dimensions of leaf and fruit. Also, an electronic balance with 0.01 g precision was used to measure the characters related to weights of fruit. The qualitative characters were recorded according to rating and coding (Table 3).

**TABLE 2:** Descriptive statistics for morphological traits utilized in the studied *Z. mauritiana* genotypes

No.	Character	Unit	Min.	Max.	Mean	*SD*	CV (%)
1	Tree growth habit	Code	1	5	2.29	1.18	51.57
2	Tree growth vigor	Code	1	5	3.44	1.17	33.98
3	Tree height	Code	1	5	3.20	1.18	36.75
4	Branching	Code	1	5	2.63	1.01	38.29
5	Branch density	Code	1	5	2.66	0.80	29.89
6	Branch flexibility	Code	1	3	2.87	0.50	17.53
7	Trunk type	Code	1	3	2.31	0.95	41.30
8	Trunk diameter	Code	1	5	2.87	0.62	21.71
9	Trunk color	Code	1	5	1.32	1.01	76.36
10	Canopy density	Code	1	5	2.53	1.10	43.32
11	Tendency to form sucker	Code	0	3	1.06	1.31	123.58
12	Leaf density	Code	1	3	2.82	0.58	20.64
13	Leaf length	mm	57.98	96.35	75.46	9.82	13.02
14	Leaf width	mm	35.02	75.95	49.54	9.52	19.22
15	Petiole length	mm	10.25	29.12	19.67	4.01	20.36
16	Petiole width	mm	1.19	2.87	1.92	0.36	18.76
17	Leaf apex shape	Code	1	3	2.24	0.97	43.48
18	Leaf upper surface color	Code	1	3	2.13	1.00	46.76
19	Leaf lower surface color	Code	1	3	2.33	0.95	40.73
20	Leaf shape	Code	1	1	1.00	0.00	0.00
21	Leaf serration shape	Code	1	3	1.32	0.74	55.76
22	Leaf serration depth	Code	1	1	1.00	0.00	0.00
23	Leaf pubescence	Code	1	1	1.00	0.00	0.00
24	Shoot spine presence	Code	1	1	1.00	0.00	0.00
25	Ripening date	Date	Mid‐Feb	Mid‐March	3.42	1.38	40.20
26	Fruit density	Code	1	5	3.05	1.57	51.54
27	Fruit shape	Code	1	5	3.57	1.77	49.55
28	Fruit length	mm	28.36	52.35	38.90	4.97	12.78
29	Fruit width	mm	26.29	43.56	33.76	3.69	10.92
30	Fruit stalk length	mm	6.35	22.32	11.75	4.32	36.79
31	Fruit stalk diameter	mm	0.97	1.68	1.33	0.18	13.60
32	Fruit weight	g	15.68	33.62	24.17	4.89	20.22
33	Fruit skin ground color	Code	1	9	5.82	2.01	34.48
34	Fruit skin over color	Code	1	9	4.16	2.95	70.96
35	Fruit taste	Code	1	7.00	5.62	1.91	33.97
36	Fruit flesh firmness	Code	1	5	3.72	1.30	34.81
37	Fruit flesh thickness	mm	9.94	17.69	13.71	1.44	10.50
38	Flesh on fruit stone	Code	1	1	1.00	0.00	0.00
39	Fruit stone length	mm	15.68	27.45	20.95	2.54	12.12
40	Fruit stone width	mm	8.18	13.54	10.03	1.39	13.86
41	Fruit stone thickness	mm	7.85	13.02	9.74	1.36	13.94
42	Fruit stone weight	g	0.76	2.52	1.52	0.53	34.50
43	Fruit stone shape	Code	1	5	3.47	1.13	32.45
44	Fruit stone surface	Code	1	1	1.00	0.00	0.00

**TABLE 3:** Frequency distribution for the measured qualitative morphological characters in the studied *Z. mauritiana* genotypes

Character	Frequency (no. of genotypes)					
	0	1	3	5	7	9
Tree growth habit	‐	Weeping (49)	Spreading (63)	Open (7)	‐	‐
Tree growth vigor	‐	Low (10)	Intermediate (73)	High (36)	‐	‐
Tree height	‐	Low (15)	Intermediate (77)	High (27)	‐	‐
Branching	‐	Low (28)	Intermediate (85)	High (6)	‐	‐
Branch density	‐	Low (21)	Intermediate (97)	High (1)	‐	‐
Branch flexibility	‐	Low (8)	Intermediate (111)	‐	‐	‐
Trunk type	‐	Single‐trunk (41)	Multitrunk (78)	‐	‐	‐
Trunk diameter	‐	Low (10)	Intermediate (107)	High (2)	‐	‐
Trunk color	‐	Gray (107)	Gray‐brown (5)	Dark Gray (7)	‐	‐
Canopy density	‐	Low (35)	Intermediate (77)	High (7)	‐	‐
Tendency to form sucker	Absent (63)	Low (21)	High (35)	‐	‐	‐
Leaf density	‐	Low (11)	Intermediate (108)	‐	‐	‐
Leaf apex shape	‐	Blate (45)	Acute (74)	‐	‐	‐
Leaf upper surface color	‐	Green (52)	Dark green (67)	‐	‐	‐
Leaf lower surface color	‐	Light green (40)	Silver‐green (79)	‐	‐	‐
Leaf serration shape	‐	Serrate (100)	Crenate (19)	‐	‐	‐
Ripening date	‐	Mid‐Feb (18)	Early‐March (58)	Mid‐March (43)	‐	‐
Fruit density	‐	Low (35)	Intermediate (46)	High (38)	‐	‐
Fruit shape	‐	Globose (34)	Ovate (17)	Ellipsoid (68)	‐	‐
Fruit skin ground color	‐	Light green (6)	Green (20)	Cream (19)	Yellow (67)	Orange (7)
Fruit skin over color	‐	Green (41)	Cream (19)	Yellow (30)	Orange (7)	Red (22)
Fruit taste	‐	Sour (6)	Sour‐sweet (23)	Slightly sweet (18)	Sweet (72)	‐
Fruit flesh firmness	‐	Low (11)	Intermediate (54)	High (54)	‐	‐
Fruit stone shape	‐	Ellipsoid (8)	Ovate (75)	Elongated (36)	‐	‐

### 2.3 Data analysis

Analysis of variance (ANOVA) was performed using SAS software (SAS Institute, Cary, NC, & USA, 1990). Simple correlations between the traits were done using Pearson correlation coefficient with SPSS software (PSS Inc., Chicago, IL, USA; Norusis, 1998). Principal component analysis (PCA) was used to study the relationships among the genotypes with SPSS software. Hierarchical cluster analysis (HCA) was performed using Ward's method and Euclidean distance with PAST software (Hammer et al., 2001). Also, the scatter plot was created using the first and second principal components (PC1/PC2) with PAST software.

## 3 RESULTS AND DISCUSSION

### 3.1 Description of characteristics

Considerable diversity was observed among the studied genotypes, as revealed by the characteristics recorded. The tendency to form sucker exhibited the greatest CV (123.58%). Also, trunk color (76.36%), fruit skin over color (70.96%), leaf serration shape (55.76%), tree growth habit (51.57%), and fruit density (51.54%) showed high CVs. Besides, the CV was more than 20.00% in the majority of characters recorded (63.36% of characters) and thus revealed significant diversity within the germplasm studied (Table 2). In contrast, six characters did not show differences, including leaf shape (lanceolate), leaf serration depth (low), leaf pubescence (present), shoot spine (present), fruit stone flesh (present), and surface of fruit stone (coarse), and thus, they can be considered as stable traits. Sharif et al. (2019) studied *Z. mauritiana* genotypes from Pakistan and reported that canopy density, color leaf lower surface, color of leaf upper surface, leaf shape, petiole length, fruit shape, fruit weight, and fruit stone shape showed high CVs.

The tree growth habit was variable and included weeping (49 genotypes), spreading (63), and opening (7). Sharif et al. (2019) studied the growth habit of *Z. mauritiana* genotypes from Pakistan and observed that tree growth habit was spreading in the most of genotypes. Also, the majority of studied genotypes exhibited moderate values for other tree‐related characters (Table 3). Gupta (2018) studied *Z. mauritiana* from Punjab in India and reported high variation in the tree‐related traits in the germplasm investigated.

Two types of leaf apex shape were recorded, including blate (45 genotypes) and acute (74). Leaf serration shape was predominantly serrated (100 genotypes). The range of leaf length was from 57.98 and 96.35 mm, while the range of leaf width was between 35.02 and 75.95 mm. Petiole length and width ranged from 10.25 to 29.12 mm and 1.19 to 2.87 mm, respectively (Table 2). Gupta (2018) studied *Z. mauritiana* from Punjab in India and reported the range of 74.20–130.00 mm for leaf length, 56.00–87.70 mm for leaf width, and 14.70–31.70 mm for petiole length. Also, Sharif et al. (2019) studied *Z. mauritiana* genotypes from Pakistan and reported the range of 18.00–91.00 mm for leaf length and 14.00–66.00 mm for leaf width. The variability in the tree‐ and leaf‐related characters is mainly due to inherent characteristics (Gupta, 2018).

The ripening date ranged from mid‐February to mid‐March. Fruit density was low in 35, intermediate in 46, and high in 38 genotypes. Fruit shape was predominantly ellipsoid (68 genotypes) (Table 3). Gupta (2018) studied *Z. mauritiana* from Punjab in India and reported high variation among the genotypes in terms of fruit shape. Sharif et al. (2019) studied *Z. mauritiana* genotypes from Pakistan and reported that round fruit shape was predominant. Fruit length and width ranged as follows: 28.36–52.35 mm and 26.29–43.56 mm, respectively. Also, fruit weighted between 15.68 and 33.62 g with an average of 24.17 (Table 2). Gupta (2018) studied *Z. mauritiana* from Punjab in India and recorded the range of 22.00–50.52 mm for fruit length, 19.40–36.20 mm for fruit width, and 5.60–27.31 g for fruit weight. Also, Sharif et al. (2019) studied *Z. mauritiana* genotypes from Pakistan and recorded the range of 8.83–48.86 mm for fruit length, 11.03–41.14 mm for fruit width, and 1.88–38.45 g for fruit weight. Fruit flesh thickness ranged from 9.94 to 17.69 mm, and fruit flesh percentage varied from 89.87 to 96.81%. Gupta (2018) recorded the range of 91.81–95.94% for fruit flesh percentage in the studied *Z. mauritiana* from Punjab in India.

Fruit skin ground color showed high variability and was predominantly yellow (67 genotypes). Gupta (2018) observed the range of yellowish‐green to light brown in *Z. mauritiana* from Punjab in India. Sweet taste (72 genotypes) was predominant in the germplasm. The firmness of fruit flesh was high and intermediate in 54 and 54 genotypes, respectively, while it was low only in 11 genotypes. Sharif et al. (2019) studied *Z. mauritiana* genotypes from Pakistan and reported the range of low to high for fruit flesh firmness.

**FIGURE 1:**
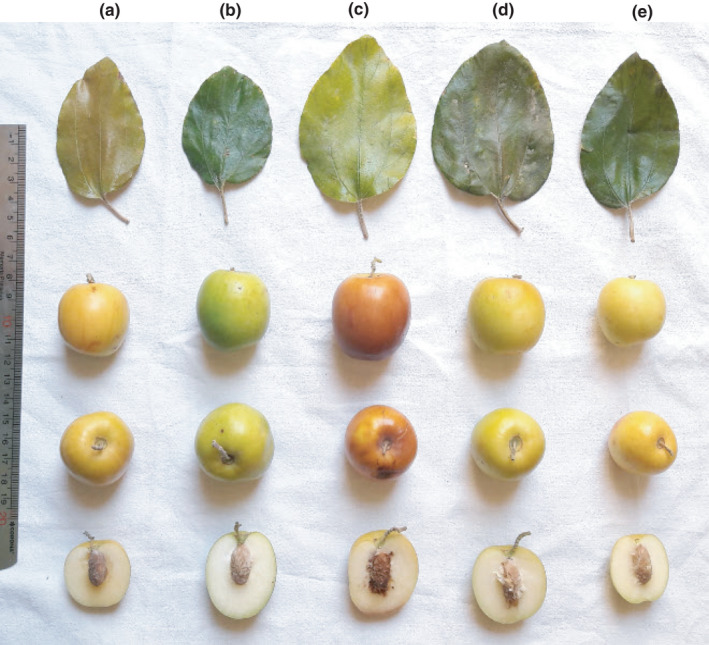
The pictures of leaves and fruits of *Z. mauritiana* genotypes studied, including a) Farzadi, b) Soopi, c) Sibi‐Ghermez, d) Sibi‐Sefid, and e) Behzadi

The mean value of length, width, and thickness of fruit stone was 20.95, 10.03, and 9.74 mm, respectively. The weight of fruit stone ranged from 0.76 to 2.52 g. Gupta (2018) recorded the range of 0.39–1.42 g for weight of fruit stone in the studied *Z. mauritiana* from Punjab in India. Also, Sharif et al. (2019) studied *Z. mauritiana* genotypes from Pakistan and observed that the weight of fruit stone was 0.32–1.84 g. Fruit stone shape was predominantly ovate (75 genotypes). Sharif et al. (2019) recorded that round fruit stone shape was predominant in *Z. mauritiana* genotypes from Pakistan. The fruit and leaf pictures of *Z. mauritiana* genotypes studied are presented in Figure 1.

### 3.2 Simple correlations between the characteristics

Pearson correlation analysis showed significant relationships between some characters (data not shown). Leaf length showed positive and significant correlations with leaf width (*r* = 0.86), petiole length (*r* = 0.63), and petiole width (*r* = 0.66) and agreed with the previous studies in *Ziziphus jujuba* (Khadivi et al., 2021; Norouzi et al., 2017) and *Z. mauritiana* (Sharif et al., 2019). Fruit density showed significant and positive correlations with tree growth vigor (*r* = 0.39), tree height (*r* = 0.25), branching (*r* = 0.31), branch density (*r* = 0.25), canopy density (*r* = 0.48), and leaf density (*r* = 0.27) and agreed with the previous studies in *Z. jujuba* (Khadivi et al., 2021; Norouzi et al., 2017) and *Z. mauritiana* (Sharif et al., 2019). Fruit weight was highly and positively correlated with fruit length (*r* = 0.54), fruit width (*r* = 0.73), fruit stalk length (*r* = 0.23), fruit stone length (*r* = 0.47), fruit stone width (*r* = 0.54), fruit stone thickness (*r* = 0.49), and fruit stone weight (*r* = 0.63) and agreed with the previous studies in *Z. jujuba* (Khadivi et al., 2021; Norouzi et al., 2017) and *Z. mauritiana* (Sharif et al., 2019).

### 3.3 PCA

**FIGURE 2:**
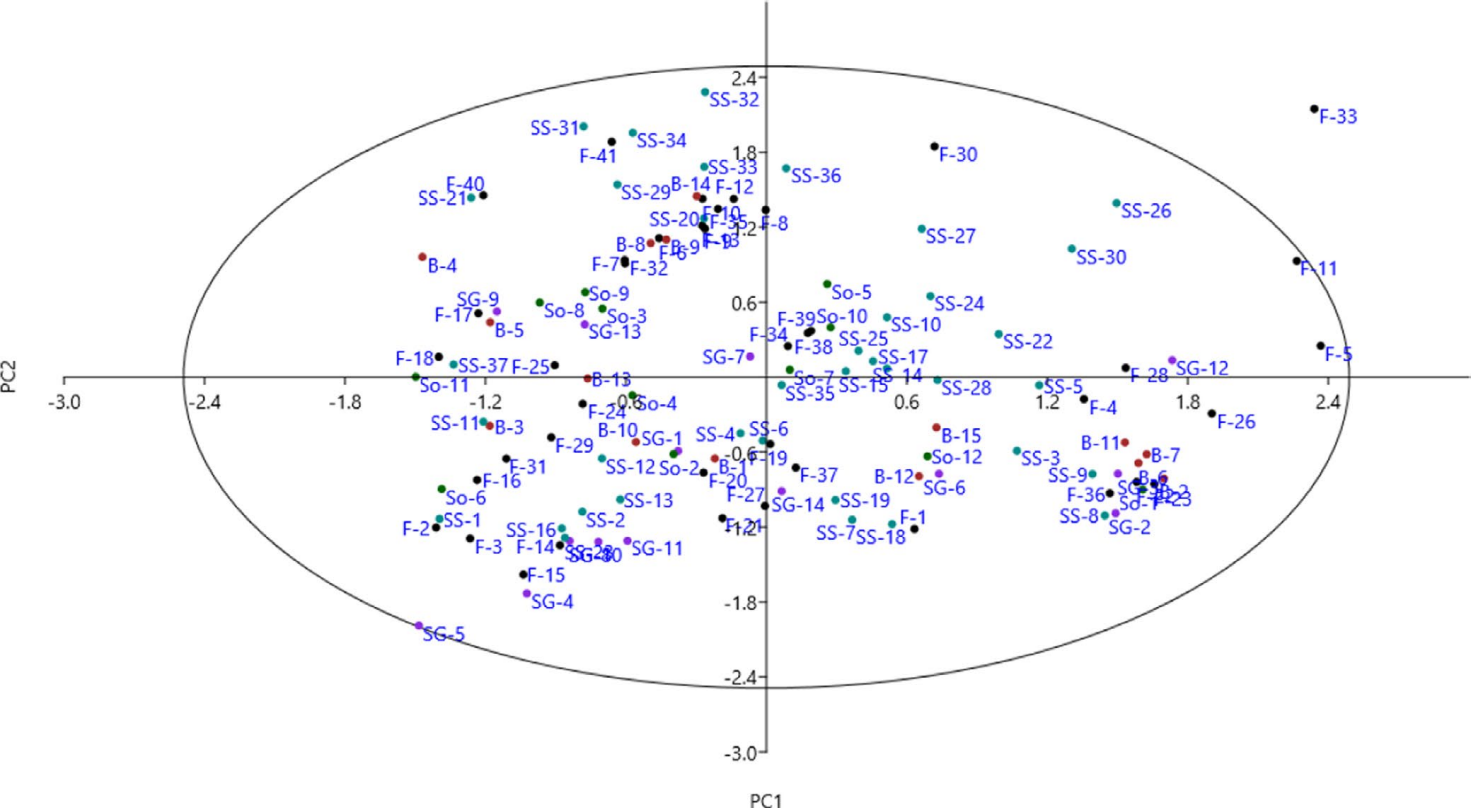
Scatter plot for the studied *Z. mauritiana* genotypes based on PC1/PC2. The symbols represent the replications of each genotype in the plot, including Farzadi (F), Sibi‐Ghermez (SG), Behzadi (B), Sibi‐Sefid (SS), and Soopi (So)

The PCA is used to find the most important traits in the data set. The purpose of the PCA is to identify a number of key components to reduce the number of characters influencing the differentiation of genotypes (Iezzoni & Pritts, 1991). Also, the relationship between the traits emphasized by this method may be consistent with the genetic link between the trait control position and the multifunctional effect (Khadivi‐Khub et al., 2014). The PCA classified the traits into 12 main components, justifying 75.07% of the total variance (Table 4). Sharif et al. (2019) studied *Z. mauritiana* genotypes from Pakistan and reported that PCA placed the characters into six PCs with explaining 82.05% of the total variance. The PC1, accounting for 16.46% of the total variance, was associated with fruit length, fruit width, fruit weight, fruit flesh firmness, fruit stone length, fruit stone width, fruit stone thickness, and fruit stone weight and agreed with the findings of Sharif et al. (2019). Six characters, including tree growth vigor, tree height, branching, branch density, canopy density, and fruit density, were placed into PC2 and accounted for 9.33% of the total variance. Four characteristics, including leaf length, leaf width, petiole length, and petiole width, were correlated with PC3, which accounted for 9.28% of the total variance. The scatter plot created using PC1 and PC2 showed the phenotypic diversity among the genotypes (Figure 2). The genotypes were distributed on the plot and were divided into two groups with four subgroups.

**TABLE 4:** Eigenvalues of the principal component axes from the PCA of morphological characters in the studied *Z. mauritiana* genotypes

Character	Component											
	1	2	3	4	5	6	7	8	9	10	11	12
Tree growth habit	0.25	−0.25	−0.25	0.10	0.13	−0.03	−0.55**	0.02	−0.07	−0.36	−0.28	−0.08
Tree growth vigor	−0.18	0.71**	0.03	−0.08	−0.29	0.05	−0.04	0.23	0.04	0.12	0.12	−0.12
Tree height	0.07	0.74**	−0.02	0.19	−0.01	0.07	−0.27	0.07	−0.14	0.06	−0.01	0.17
Branching	−0.09	0.80**	−0.06	0.02	0.09	−0.03	−0.05	−0.05	0.01	0.11	0.02	0.05
Branch density	0.01	0.73**	−0.07	0.07	−0.24	−0.03	0.27	0.02	0.03	−0.07	−0.04	0.13
Branch flexibility	−0.12	0.10	0.05	−0.07	−0.03	0.04	0.01	0.70**	0.22	0.07	0.06	−0.01
Trunk type	−0.14	0.03	−0.09	−0.12	0.35	0.02	−0.18	−0.57**	0.18	−0.02	−0.08	0.36
Trunk diameter	0.00	0.32	0.05	0.18	0.11	0.05	−0.29	0.62**	−0.09	−0.02	−0.13	0.22
Trunk color	0.10	0.00	−0.07	−0.07	0.03	0.08	0.05	0.01	−0.02	0.01	0.02	0.82**
Canopy density	−0.02	0.64**	−0.08	0.01	0.33	0.04	0.14	0.15	−0.06	−0.12	−0.08	−0.18
Tendency to form sucker	0.13	0.05	−0.05	0.00	0.09	0.12	0.89**	−0.01	−0.03	−0.07	−0.05	0.02
Leaf density	0.08	0.46	0.12	0.07	−0.30	−0.31	0.24	0.37	0.01	−0.14	−0.16	0.08
Leaf length	−0.09	0.02	0.91**	0.00	0.14	0.05	0.01	0.08	−0.07	0.14	0.02	0.02
Leaf width	−0.02	−0.07	0.89**	−0.24	0.05	0.08	0.07	0.06	−0.06	−0.03	−0.11	0.02
Petiole length	−0.11	−0.15	0.80**	0.16	−0.35	0.03	−0.01	0.03	0.05	0.02	−0.08	−0.06
Petiole width	−0.09	−0.05	0.82**	0.07	−0.11	−0.09	−0.06	−0.03	0.21	0.05	−0.02	−0.11
Leaf apex shape	0.25	0.08	−0.06	0.76**	0.07	−0.02	−0.11	0.11	0.01	0.11	−0.02	−0.08
Leaf upper surface color	0.13	−0.01	0.03	0.09	−0.26	0.27	−0.12	0.11	0.68**	0.03	0.04	0.05
Leaf lower surface color	0.03	−0.16	−0.24	0.27	−0.10	0.37	0.17	0.06	0.53	0.16	−0.17	0.23
Leaf serration shape	−0.12	−0.07	−0.06	−0.19	0.15	−0.12	0.13	0.03	0.43	−0.50	0.24	0.34
Ripening date	−0.02	−0.19	−0.05	−0.11	0.74**	−0.01	0.18	−0.10	−0.04	−0.14	−0.09	0.15
Fruit density	0.28	0.58**	−0.07	−0.14	0.07	−0.12	0.11	0.01	0.01	−0.10	−0.05	−0.24
Fruit shape	0.25	0.02	−0.06	−0.68**	0.23	0.02	−0.14	0.01	−0.08	0.23	−0.34	0.04
Fruit length	0.88**	0.03	−0.03	−0.02	−0.05	0.02	0.01	0.03	0.14	−0.04	0.10	0.03
Fruit width	0.79**	−0.07	0.05	0.21	−0.08	0.07	−0.07	−0.07	0.15	−0.21	0.31	0.02
Fruit stalk length	0.54	−0.06	0.21	−0.06	0.04	−0.09	0.01	0.07	0.09	0.63**	−0.09	0.13
Fruit stalk diameter	0.41	−0.02	0.47	−0.21	−0.03	−0.05	0.07	0.16	−0.04	0.56	0.22	0.04
Fruit weight	0.65**	0.08	−0.05	0.52	0.05	0.07	−0.09	−0.19	0.03	−0.11	0.23	−0.01
Fruit skin ground color	−0.05	0.02	0.06	−0.08	−0.01	0.86**	0.08	−0.01	0.12	−0.02	−0.07	0.02
Fruit skin over color	−0.23	0.00	−0.17	0.04	−0.14	−0.04	−0.03	0.01	−0.65**	0.08	0.10	0.14
Fruit taste	0.19	−0.04	0.02	0.07	0.01	0.85**	0.03	0.04	0.11	−0.01	0.08	0.07
Fruit flesh firmness	0.67**	−0.18	0.02	0.21	−0.34	0.03	0.06	−0.21	0.09	−0.05	−0.22	−0.15
Fruit flesh thickness	0.19	−0.05	−0.13	0.16	0.01	0.00	0.01	0.02	−0.09	−0.01	0.84**	0.02
Fruit stone length	0.82**	0.05	−0.13	0.00	0.18	−0.01	0.07	0.06	0.10	0.25	−0.08	0.15
Fruit stone width	0.95**	−0.02	−0.05	−0.07	−0.03	0.03	0.07	0.05	0.00	0.06	0.01	0.03
Fruit stone thickness	0.93**	0.03	−0.07	−0.11	0.02	0.04	0.04	0.07	−0.04	0.12	−0.02	0.01
Fruit stone weight	0.73**	0.05	−0.20	0.21	0.13	0.07	−0.12	−0.20	−0.03	0.27	0.03	−0.10
Fruit stone shape	0.04	0.16	−0.13	0.24	0.62**	0.01	−0.33	−0.02	−0.05	0.31	0.18	−0.18
Total	6.25	3.54	3.53	1.95	1.94	1.88	1.74	1.65	1.64	1.63	1.43	1.36
% of Variance	16.46	9.33	9.28	5.12	5.11	4.94	4.58	4.33	4.32	4.28	3.75	3.58
Cumulative %	16.46	25.79	35.06	40.19	45.30	50.23	54.81	59.15	63.46	67.74	71.49	75.07

Note

** Eigenvalues ≥0.55 are significant at the *p* ≤ .01 level.

### 3.4 HCA

**FIGURE 3:**
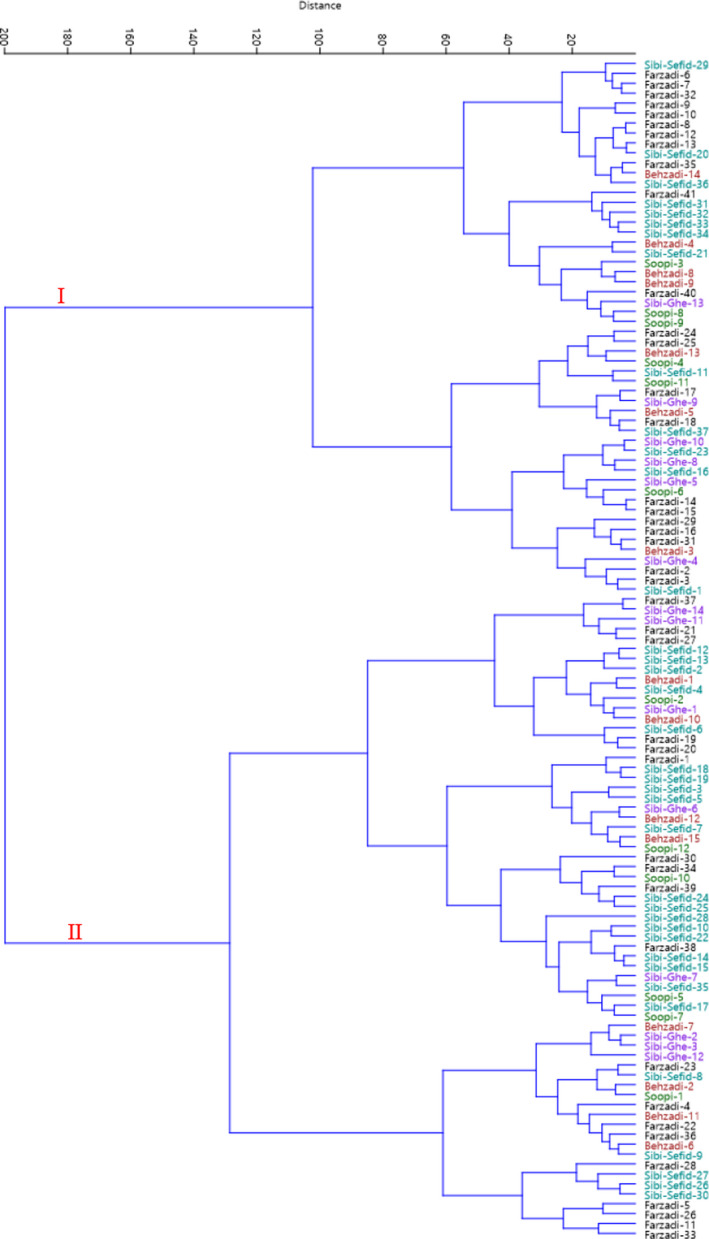
Ward cluster analysis of the studied *Z. mauritiana* genotypes based on morphological traits using Euclidean distances

**FIGURE 4:**
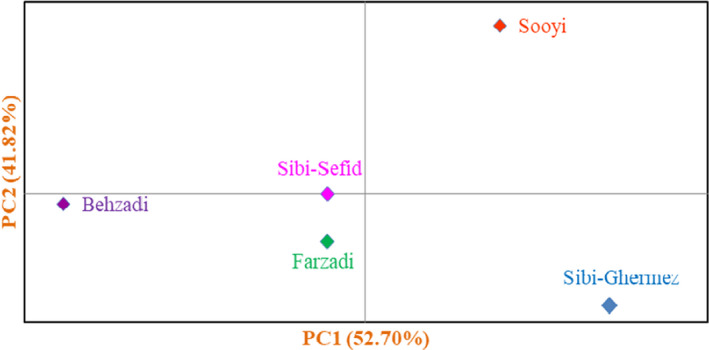
Biplot for the studied populations of *Z. mauritiana* based on morphological characters

The genotypes studied were placed into two major clusters according to all the traits using HCA (Figure 3). The first cluster (I) included two subclusters. Subcluster I‐A consisted of 27 genotypes, and subcluster I‐B contained 27 genotypes. The remaining genotypes were classified into the second cluster (II), forming two subclusters. Subcluster II‐A consisted of 43 genotypes, and subcluster II‐B contained 22 genotypes. Besides, population analysis showed that the studied populations were divided into three main groups (Figure 4). The first group included Soopi population, characterized by the lowest values for leaf length and leaf width and also the highest value for fruit weight. The second group consisted of Sibi‐Ghermez population, characterized by the lowest values for fruit length, fruit width, fruit stalk length, fruit stalk diameter, fruit weight, fruit stone length, fruit stone width, fruit stone thickness, and fruit stone weight. Also, the third group included Behzadi, Farzadi, and Sibi‐Sefid populations, characterized by high values for leaf, fruit, and stone. The genotypes with desirable values for fruit yield and fruit quality are always selected by breeder for using in breeding programs and also for cultivation (Mahmood et al., 2014). Here, phenotypic diversity was considerable among the genotypes selected, which can be used to begin the comprehensive investigations on genetic resources of *Z. mauritiana* and to select the promising genotypes for cultivation.

The diversity observed in the fruit size and quality‐related characteristics between the studied genotypes can encourage the implementation of breeding programs. The results also showed that fruit weight among different genotypes has considerable diversity. The variation in fruit weight among the genotypes grown in the same geographical areas is due to differences in genetic basis and ecological conditions (Umbreen et al., 2018). The genotypes with high fruit weight can be selected for fresh fruit production and introduction to growers. The length and width of the fruit, which are important traits for breeders, also showed considerable diversity. The study of fruit size‐related traits is of great importance for packaging and shipping judgments. Also, the color and taste of the fruits of different genotypes showed a great diversity, which can help breeders to choose genotypes according to the type of consumption (Liu et al., 2009).

## 4 CONCLUSION

The current findings can be widely used to introduce cultivars in breeding programs of *Z. mauritiana*. The present information might be used to choose the genotypes with the desired traits. Finally, 21 genotypes, including Sibi‐Sefid‐35, Farzadi‐41, Farzadi‐40, Sibi‐Sefid‐34, Sibi‐Sefid‐33, Sibi‐Sefid‐32, Behzadi‐9, Behzadi‐8, Soopi‐3, Sibi‐Sefid‐37, Soopi‐9, Farzadi‐30, Farzadi‐4, Soopi‐5, Sibi‐Ghermez‐13, Sibi‐Sefid‐10, Sibi‐Sefid‐22, Farzadi‐12, Soopi‐7, Sibi‐Sefid‐30, and Sibi‐Ghermez‐7, were promising because of high values for fruit weight, fruit taste, fruit skin color, and fruit quality that might be directly cultivated and used in breeding programs. Besides, the genotypes with superior traits can be further used for improvement through selection and hybridization to get desired traits.

## CONFLICT OF INTEREST

The authors declare no conflict of interest.

## AUTHOR CONTRIBUTIONS

**Farhad Mirheidari:** Investigation (equal). **Ali Khadivi:** Formal analysis (lead), Methodology (lead), Project administration (lead), Supervision (lead), Writing – review & editing (lead). **Abdolvahid Saeidifar:** Investigation (equal). **Younes Moradi:** Investigation (equal).

## RESEARCH INVOLVING HUMAN PARTICIPANTS AND/OR ANIMALS

None.

## INFORMED CONSENT

None.

## Data Availability

The data that support the findings of this study are available from the corresponding author upon reasonable request.
